# Exploiting parallelization in positional Burrows–Wheeler transform (PBWT) algorithms for efficient haplotype matching and compression

**DOI:** 10.1093/bioadv/vbad021

**Published:** 2023-03-02

**Authors:** Rick Wertenbroek, Ioannis Xenarios, Yann Thoma, Olivier Delaneau

**Affiliations:** School of Engineering and Management Vaud (HEIG-VD), HES-SO University of Applied Sciences and Arts Western Switzerland, Yverdon-les-Bains 1401, Switzerland; Department of Computational Biology, University of Lausanne, Lausanne 1015, Switzerland; Department of Computational Biology, University of Lausanne, Lausanne 1015, Switzerland; School of Engineering and Management Vaud (HEIG-VD), HES-SO University of Applied Sciences and Arts Western Switzerland, Yverdon-les-Bains 1401, Switzerland; Department of Computational Biology, University of Lausanne, Lausanne 1015, Switzerland

## Abstract

**Summary:**

The positional Burrows–Wheeler transform (PBWT) data structure allows for efficient haplotype data matching and compression. Its performance makes it a powerful tool for bioinformatics. However, existing algorithms do not exploit parallelism due to inner dependencies. We introduce a new method to break the dependencies and show how to fully exploit modern multi-core processors.

**Availability and implementation:**

Source code and applications are available at https://github.com/rwk-unil/parallel_pbwt.

**Supplementary information:**

Supplementary data are available at *Bioinformatics Advances* online.

## 1 Introduction

The positional Burrows–Wheeler transform (PBWT) data structure allows for the development of efficient matching algorithms between haplotypes (Durbin, 2014). That is, the PBWT allows matching in linear time relative to the number of haplotypes instead of the quadratic time of a naive all-versus-all approach. Another advantage is the impressive data compression rate made possible by this data structure. This makes the PBWT, and associated algorithms, a core component of bioinformatics tools, such as *Beagle*, *EagleImp*, *GLIMPSE* or *XSI* (Browning *et al.*, 2021; Rubinacci *et al.*, 2021; Wertenbroek *et al.*, 2022; Wienbrandt and Ellinghaus, 2022), and bioinformatics courses (Gagie *et al.*, 2022). However, algorithms relying on the PBWT for processing haplotypes over a genomic region exhibit a positional dependency, i.e. the state of the structure at a given position (genomic locus) depends on the previous position. This makes it hard to parallelize. Others (Sanaullah *et al.*, 2021; Shakya *et al.*, 2022; Wang *et al.*, 2023) have proposed methods to make PBWT algorithms more efficient but are still sequential. In this article, we introduce a method to manage the dependency and split the problem for parallel execution. We show that with this new method haplotype matching algorithms can achieve a speed-up of up to 8× on a modern 12-core processor.

## 2 Methods

The algorithms presented in Durbin (2014) for haplotype matching and compression rely on two key internal data structures: the positional prefix array *a_k_* which represents the ordering of haplotypes at position *k* and the divergence array *d_k_* which stores the position where a haplotype differs from the previous one in the current ordering. (For definitions (see Durbin, 2014), the same nomenclature is used here). The *a_k_* and *d_k_* arrays for a position *k* are built from the arrays of the previous position $a_{k-1}$ and $d_{k-1}$. This dependency propagates back until the initial arrays *a*_0_ and *d*_0_ which are given. The positional prefix array *a*_0_ represents the arbitrary order the haplotypes come in from the input data, at each position the haplotypes are reordered given the genotype they carry at that position, either a 0 (reference genotype) or a 1 (alternative genotype). So when generating *a*_1_ all haplotypes with the reference genotype (0) at the first position (*k *=* *0) come before those that have an alternate genotype (1) at position *k *=* *0. This is equivalent to a *radix sort* (also known as *digital sort* or *bucket sort*). Therefore, at each position *k*, the *k* previous genotypes (reverse prefix) dictate the position of that haplotype. The divergence array *d_k_* is generated at the same time by keeping track of at which position *k*, previously matching haplotypes stop matching (do not share the same genotype anymore). These two arrays are key in PBWT-based algorithms for matching or compression. To start processing at an arbitrary position *k* the arrays *a_k_* and *d_k_* must be known (iteratively computed from the starting position 0 up to *k*). This dependency makes it difficult to split a genomic region with *N* loci, k∈[0,N[ between separate threads for parallel processing.

### 2.1 Splitting the genomic region and breaking the dependency

A key observation is that if we generate *a_k_* and *d_k_* from a previous position *k − b* (over a chunk of *b* previous loci, *b *<* k*) with initial arrays *a*_0_ and $d\prime_{k-b}$ (an array filled with the value *k − b*) instead of the actual arrays $a_{k-b}$ and $d_{k-b}$, then for all haplotypes, except the ones that are identical over *k − b* to *k*, the computed values in the *a_k_* and *d_k_* arrays will be correct. That is, as if computed from the start, i.e. starting at *k *=* *0 with *a*_0_ and *d*_0_ and iterating over k loci instead of only *b* loci. This means we have an approximated version of *a_k_* and *d_k_* that can only have wrong values for groups of identical haplotypes over the chunk of *b* loci. For *a* because they cannot be ordered given the *b* observed loci and for *d* because they differ at a loci before *k − b*. Because $d\prime_{k-b}$ was initialized with *k − b* the condition $d_{k}[i]=k-b$ lets us know for which indices *i* the values of *a_k_* and *d_k_* might be wrong. **Keypoint:** The correction of arrays *a_k_* and *d_k_* computed from position *k − b* (*b* steps) with *a*_0_ and $d\prime_{k-b}$ instead of $a_{k-b}$ and $d_{k-b}$ can be done in a single step if the correct $a_{k-b}$ and $d_{k-b}$ are known. **Strategy:** It is possible split the genomic region of *N* loci into *t* chunks of b=N/t loci for *t* threads to handle in parallel. Each thread can compute the approximated *a_k_* and *d_k_* arrays (end of the chunk) from the position *k − b* (start of the chunk), with *a*_0_ and $d\prime_{k-b}$ instead of the actual arrays for the start of each chunk. The *a_k_* and *d_k_* arrays at the end of the first chunk will be correct because the first chunk is supposed to start with *a*_0_ and *d*_0_ (note, $d\prime_{0}=d_{0}$). Then, we can use the *keypoint* above to correct the remaining *t −* 1 *a* and *d* arrays in *t −* 1 sequential steps. Because *t* will typically be small (e.g. 2–64 threads), the number of steps executed sequentially is small compared to the total number of steps *N* (e.g. in the millions). Also, the bigger the chunk the smaller the chance to have identical haplotypes, the less time will be required to correct the *a* and *d* arrays. Once the *t* arrays are generated the heavier algorithms (matching, compression, etc.) can be launched in parallel with *t* threads. The process is illustrated in the Supplementary Figures SP1–SP4.

### 2.2 Algorithms to correct approximated *a* and *d* arrays

The method to correct *a* and *d* is decomposed into two algorithms; Algorithm 1 shows how to fix *a_k_* and *d_k_*, between a *start* and *stop* index, given the arrays at *k − b*. The start and stop indices represent a group of identical haplotypes over loci *k − b* to *k*. To rearrange the haplotypes in *a_k_* between *start* and *stop*, they require to follow the order given in $a_{k-b}$. To do so, the positions of the haplotypes in the previous chunk are looked up in $a_{k-b}^{-1}$, which is the inverse of the positional prefix array $a_{k-b}$ (see Algorithm 2). These positions are then sorted in incremental order and finally the correct order is set in *a_k_* by referring to the haplotype number in $a_{k-b}$ given the incrementally sorted indices.

Now that the group of identical haplotypes are ordered correctly (correct values in *a_k_*), we need to fix the divergence values, the first value at position *start* will already be correct because it refers to the previous non-matching haplotype. For the other values, they now need to be updated to reflect the divergences in the previous chunk given the corrected ordering. Although the haplotypes are grouped together in the current chunk, they might have other haplotypes in between them in the previous chunk. This requires to scan for the biggest value of *d* between the previous haplotype and the current one referring to the ordering in the previous chunk, similarly to what is done with *p*, *q* of Algorithm 2 presented in Durbin (2014).

**Algorithm 1:** Correction of positional prefix and divergence arrays *a_k_*, *d_k_* between *start* and *stop* given previous arrays $a_{k-b},\;d_{k-b}$

Initialization, create array arr[stop−start]

**for**i:=start*to stop − 1* **do**

 $\mathrm{arr}[i-\mathrm{start}]:=a_{k-b}^{-1}[a_{k}[i]]$

**end**

sort (*arr*)

**for**i:=start*to stop − 1* **do**

 $a_{k}[i]:=a_{k-b}[\mathrm{arr}[i-\mathrm{start}]]$

**end**

**for**i:=start+1*to stop − 1* **do**

 scan_start:=arr[i-start-1]+1

 scan_stop:=arr[i-start]+1

 $d_{k}[i]=\text{max}\_\text{element}(d_{k-b}[\text{scan}\_\text{start}:\text{scan}\_\text{stop}])$

**end**

Algorithm 2 allows to determine groups of matching haplotypes over *k − b* to *k* and calls Algorithm 1 to fix the values in *a_k_* and *d_k_*. To do so Algorithm 2 has to generate the array $a_{k-b}^{-1}$, which is required for Algorithm 1. This is done by looping through all entries of $a_{k-b}$ which are haplotype identifiers, so if $a_{k-b}[0]=id_{x}$ it means the first haplotype is *id_x_*, therefore $a_{k-b}^{-1}[id_{x}]=0$. So *a* maps positions to identifiers and $a^{-1}$ maps identifiers to positions. Then Algorithm 2 iterates over the *d* array keeping track of haplotype groups matching over *k − b* to *k*, for each of such groups it will call Algorithm 1 to correct the *a* and *d* arrays.

Algorithm 2 is used to sequentially correct the approximate *a* and *d* arrays generated in parallel with the strategy proposed in Section 2.1 (see Algorithm P in Section 2.3). A step-by-step example of the execution of Algorithms 1 and 2 is provided in the Supplementary Materials and illustrated with Supplementary Figures S1 and S2.

**Algorithm 2:** Correction of positional prefix and divergence arrays *a_k_*, *d_k_* given correct previous arrays $a_{k-b},\;d_{k-b}$

initialization, create array $a_{k-b}^{-1}[M]$, group_index: = 0

**//** Fill $a_{k-b}^{-1}$ array, reciprocal of $a_{k-b}$ array

**for** i:=0 *to M − 1* **do**

 $a_{k-b}^{-1}[a_{k-b}[i]]:=i$

**end**

// Iterate over the divergence array to find and fix matching groups **for**i:=0*to M − 1* **do**

 **if**$d_{k}[i]\neq p$**then**

  **if**i−group_index>1**then**

   **Algorithm 1** with start := group_index and stop :=i

  **end**

  group_index: = *i*

 **end**

**end**

**if** M−group_index>1 **then**

 **Algorithm 1** with start := group_index and stop :=M

**end**

### 2.3 From sequential to parallel algorithm

The sequential PBWT-based algorithms can be summarized with Algorithm S which loops over all *N* genotype loci and alternates between updating the *a* and *d* arrays and running the matching algorithm or compression step.

**Algorithm S**: Sequential PBWT-based algorithm

**Constants**: *N*: #genotype loci, *M*: #haplotypes

Initialization, create array $a_{0}=[0,1,2,\ldots ,M-1]$ and $d_{0}=[0,\ldots ,0]$

**for**k:=0*to N* **do**

 Run matching [e.g. Algorithm 3 or 4 from Durbin (2014)] or compression step

 Generate $a_{k+1}$ and $d_{k+1}$ from *a_k_* and *d_k_*

**end**

Our parallel implementation is described in Algorithm P. The algorithm relies on the *keypoint* and *strategy* presented above. The algorithm starts by a parallel step to generate the approximate *a* and *d* arrays, then runs a small sequential loop to correct these arrays with Algorithm 2 presented above. Finally, it launches *T* threads that will each run Algorithm S with the heavier matching or compression algorithms. Each thread handles a chunk of the genomic region starting at positions k∈{0,b,2b,3b,…,(T−1)·b} with the now available and correct *a_k_*, *d_k_* arrays (instead of a single thread running Algorithm S over the whole genomic region starting at *k *=* *0 with *a*_0_ and *d*_0_ and finishing at *N*).

**Algorithm P**: Parallel PBWT-based algorithm

**Constants**: *N*: #genotype loci, *M*: #haplotypes, *T*: #threads

b=N/T //Chunk size

// **A**: Parallel generation of approximate *a* and *d* arrays

Launch *T −* 1 threads with Algorithm 2 from Durbin (2014) for *b* steps starting from positions k∈{0,b,2b,3b,…,(T−2)*b} with arbitrary $a_{k}=[0,1,2,\ldots ,M-1]$ and $d_{k}=[k,\ldots ,k]$

that will generate the approximate *a_i_* and *d_i_* arrays (with i∈{b,2b,3b,…,(T−1)*b})

// **B**: Sequential correction of the approximate arrays *a_i_*, *d_i_*

**for** t:=1 *to T − 1* **do**

 join(thread *t*)

 **if** *t > 1 // (Note: a_b_, d_b_ are already correct)* **then**

  **Algorithm 2**: correct $a_{t\cdot b}$ and $d_{t\cdot b}$ with $a_{(t-1)\cdot b}$ and $d_{(t-1)\cdot b}$

 **end**

**end**

// **C**: Parallel run of matching Algorithm 3 or 4

Launch *T* threads running **Algorithm S** with e.g. Algorithm 3 or 4 from Durbin (2014) for *b* steps starting from positions k∈{0,b,2b,3b,…,(T−1)*b} with the now available and correct *a_k_*, *d_k_* arrays

### 2.4 Time and space complexity analysis

The worst case time complexity of Algorithm 1 relative to *M* input haplotypes (if all haplotypes match from *k − b* to *k*, with *a* and *d* that require to be corrected) is quasilinear, O(M log M). Algorithm 1 can be split in four steps, three loops and one sorting algorithm: The first two loops iterate over the matching haplotypes so they are O(M). The sort is O(M log M) because it is implemented with a merge sort (Knuth, 1975). The last loop may look like it could have quadratic complexity because of the inner *max element* look-up, but it has not. The number of look-ups for the combined *max elements* is bounded by the number of haplotypes, because the array *arr* is comprised of sorted positions we have 0≤scan_start<scan_stop≤M and $\sum_{i}\left(\text{scan}\_\text{stop-scan}\_\text{start}\right)$ is bounded by *M*, therefore the number of look-ups in this loop is O(M).

The worst case time complexity of Algorithm 2 relative to *M* input haplotypes is quasilinear O(M log M). Algorithm 2 has two main parts: first, it generates $a_{k-b}^{-1}$ which is done in *M* steps, therefore it is O(M). Second, it has to apply Algorithm 1 to a number of matching haplotype groups. The time complexity of Algorithm 1 is dominated by the sorting step which is O(M log M). The worst case time complexity of the second part of Algorithm 2 is also O(M log M), because either all haplotypes match and we have a single group of size *M* to sort (apply Algorithm 1), or we have a given number of smaller groups to sort. Because the sum of the group sizes is bounded by *M*, running a number of smaller sorts will require a lower or equal asymptotic number of steps than sorting all the haplotypes (e.g. apply Algorithm 1 to *M* groups of size 1). Therefore, the worst case time complexity of running Algorithm 2 is O(M log M).

The space complexity is O(M) because a constant number of arrays of size *M* is required and the merge sort also has linear space complexity (Knuth, 1975).

The PBWT algorithms for matching and compression have a worst case time complexity of O(NM) where *N* is the number of genotype loci and *M* the number of haplotypes. Our parallel version will have a worst case time complexity of O(NM/T+TM log M) where *T* is the number of threads. The added O(TM log M) quasilinear complexity is negligible compared to the gains we have by dividing *NM* by *T*. For example, with data from (1000 Genomes Project Consortium and others, 2015): N≥88,000,000, *M *=* *5008, and *T* will typically be small (e.g. 2–64 threads).

## 3 Results

**Fig. 1.**
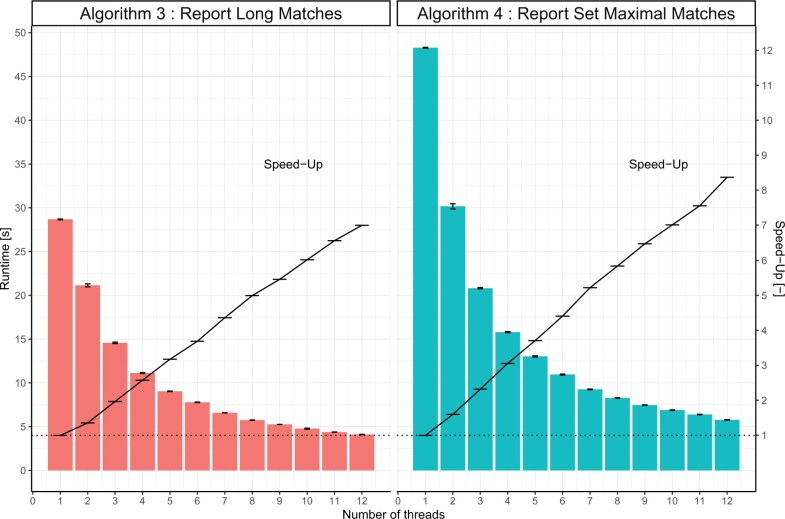
Runtime and speed-up of Algorithms 3 and 4 from Durbin (2014) and their parallel implementations running on a 12-core AMD 3900X processor (each run 10 times). Algorithm 3 reports all matches longer than 2000 genotypes (loci) between all haplotypes, Algorithm 4 reports the set maximal matches between all haplotypes. Data are chromosome 20 from (1000 Genomes Project Consortium and others, 2015) 5008 haplotypes, 1 822 268 genotype loci

We applied the strategy above on two haplotype matching algorithms from Durbin (2014) and implemented them as the parallel implementation shown in Algorithm P: Algorithm 3 which reports all matches between haplotypes above a given length and Algorithm 4 which reports all *set-maximal* matches between haplotypes. Figure 1 shows the runtime of the original single-threaded algorithm (Algorithm S) and its multi-threaded counterparts (Algorithm P) for different number of threads *t* on data from (1000 Genomes Project Consortium and others, 2015). With an AMD 3900X processor. The multi-threaded implementations achieve a speed-up of 7× and 8.37× on Algorithms 3 and 4 respectively running with 12 threads. Results on the Human Reference Consortium data (McCarthy *et al.*, 2016) are available in the Supplementary Materials (with a similar speed-up of 7.04× and 8.16× for 12 threads), as well as an example application that implements the matching algorithms and reports the results to a file to allow a direct comparison to the original software from Durbin (2014). The Supplementary Materials also provide a comparison between generating the *a* and *d* arrays sequentially with Algorithm 2 from Durbin (2014) against our parallel implementation with sequential correction (Algorithms 1 and 2) presented here (Sections A and B of Algorithm P) for different number of threads. Supplementary Figure SP5 shows that the parallel version followed by the sequential correction can provide a speed-up of up to 10.94× when generating the *a* and *d* arrays.

## 4 Discussion

In this article, we have presented a method and two algorithms that allow parallel execution of PBWT-based haplotype matching algorithms. The method allows to exploit modern multi-core processors and has shown a 7×–8.37× reduction in execution time with 12 threads compared to the single-threaded version. For PBWT-based compression, some methods break the per loci dependency by design for better random access performance (e.g. Wertenbroek *et al.*, 2022). Therefore, these algorithms can be multi-threaded directly. However, compression methods that do not break this dependency (e.g. Deorowicz and Danek, 2019; Durbin, 2014; LeFaive *et al.*, 2021; Li, 2016) could be accelerated by the presented methods. Beside these results, the *a* and *d* arrays could be saved to a file so that subsequent runs of the algorithms could be launched in parallel directly and now these arrays can be generated efficiently in parallel thanks to Algorithms 1 and 2 presented here, with up to a 10.94× reduction in time. Algorithm 2 also exhibits another interesting property: it provides the indices of groups of haplotypes that are identical over a large genomic chunk. This information could be leveraged to speed up PBWT-based methods, e.g. (Hofmeister *et al.*, 2022), by treating the whole group as a single haplotype block and avoid redundant computations.

## Supplementary Material

vbad021_Supplementary_Data
