# Supplementary material for: Exploiting parallelization in positional Burrows–Wheeler transform (PBWT) algorithms for efficient haplotype matching and compression
Source: Bioinform Adv. 2023 Mar 2;3(1):vbad021. doi: 10.1093/bioadv/vbad021 (PMC10005600; doi:10.1093/bioadv/vbad021)
Supplement: vbad021_Supplementary_Data [file vbad021_supplementary_data.pdf]

Exploiting parallelization in positional Burrows-Wheeler transform (PBWT) algorithms - Supplementary Materials

Rick Wertenbroek, Ioannis Xenarios, Yann Thoma, and Olivier Delaneau

Positional Burrows-Wheeler Transform

The dependency

The PBWT rearranges haplotypes at each position (variant locus) given their reversed-prefix order, as shown in Fig. S1. In order to do this rearrangement (sort) efficiently for each position the arrangement of the previous position is required. This is equivalent to a 1-bit radix sort. To make the algorithm memory efficient the rearrangements (positional prefix array) "**a**" and divergence array "**d**" (leftmost position where the sequence matched) are not saved for every position, and only the arrays for the current position are kept. This makes each step of the algorithm dependent on the previous step. This inner dependency makes the parallelization of the algorithm intricate. Fig. S1 Shows the update of the positional prefix array "**a**" and divergence array "**d**" for 10 example haplotypes with 10 loci.

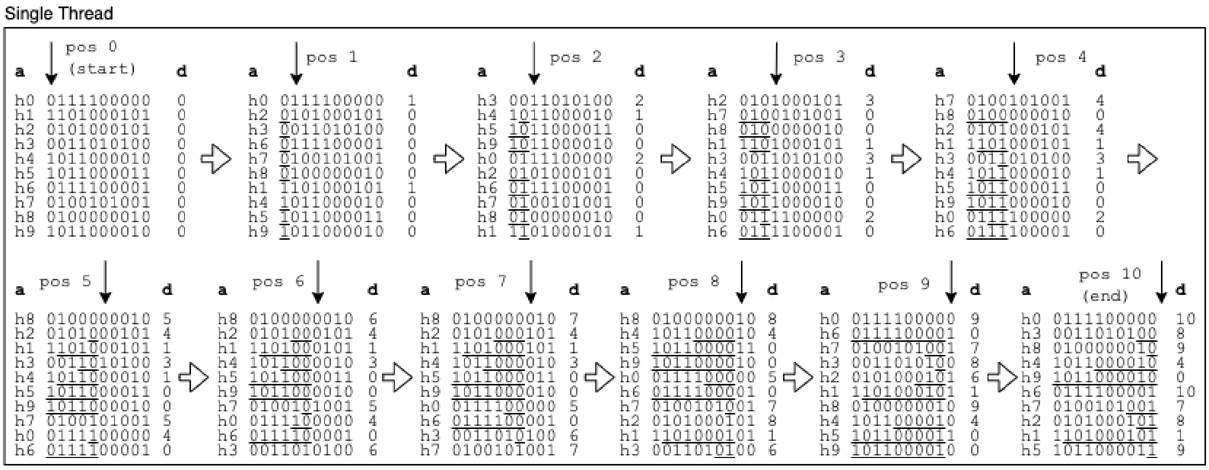

Fig. S1 Shows the subsequent arrangements of 10 haplotypes (h0-h9) through the steps of the PBWT algorithm. The arrangement of the given step is shown in array **a** (positional prefix array). For each haplotype the genotype data (0-ref. allele, 1-alt. allele) is shown on the corresponding line. The **d** (divergence) array reflects the position where a match starts between an haplotype and the one just above it (shown as underlined values).

Positional Burrows-Wheeler Transform with parallel generation of **a** and **d** arrays and algorithms 1-2

As an example the same data set as in Fig. S1 is used here. For the example we want to generate the a and d arrays for positions 5 and 10 in parallel. Two threads are launched, one from the start (position 0) and one from position 5. They both have 5 steps to perform, as illustrated in Fig. S2 and both start from an arbitrary arrangement of the haplotypes (h0-h9).

When both threads finish execution, we have the correct arrays a and d for position 5 but the arrays for position 10 may be wrong because thread 2 started at position 5 with an arbitrary arrangement of haplotypes (instead of the one generated by thread 1). Now that both threads generated the arrays in parallel, we can use algorithm 1 and 2 of this paper to fix the arrays of position 10 with those of position 5 (k = 10, p = 5).

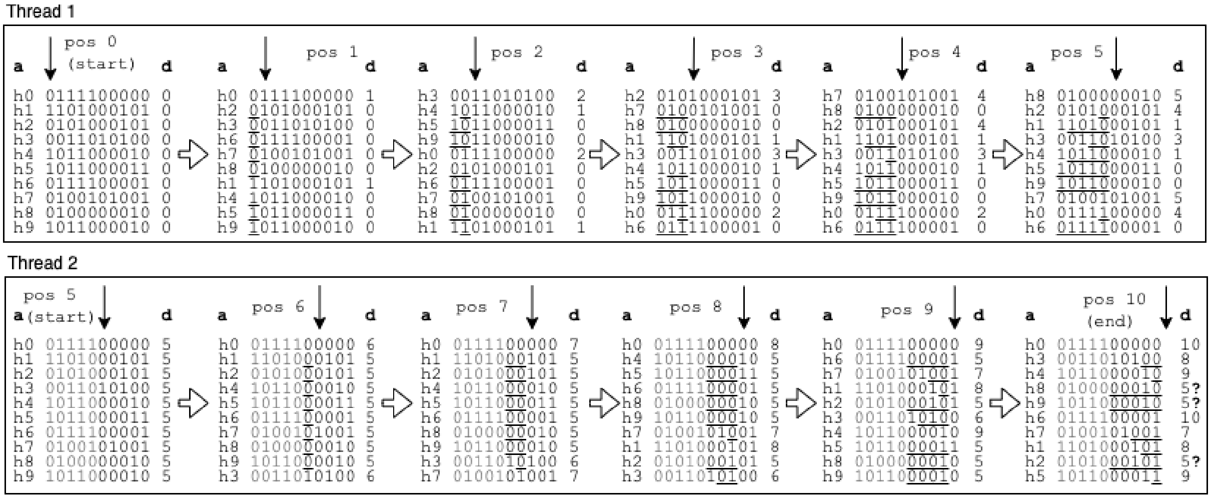

Fig S2. Two threads compute the **a** and **d** arrays from arbitrary ordering in parallel. Thread 1 from the start (position 0) and Thread 2 from position 5. Once the computation is done, the arrays computed by Thread 1 for position 5 are now known and can be used to fix the arrays generated by Thread 2. The order between h4,8,9 and h1,2 at position 10 may be erroneous because the haplotypes are the same from position 5 to 10 (10 not included). The divergences in **d** marked with ? must be verified and possibly corrected with algorithm 1 and 2.

$\mathbf{d}_{10}$ is traversed with algorithm 2. The first value, 10 is different from 5 and the previous group is of size 0 so we continue, same for 8 and 9 but the previous groups are of size 1. Then we encounter 5 twice and therefore group them. When we encounter 10 for h6 we check the size of the previous group, which is 3 (haplotypes h4,8,9) and since it is bigger than 1 we have to check the arrangement and divergence (for this group; start = 2, stop = 5). Algorithm 1 is used to fix the values as described below.

A look-up in $\mathbf{a}^{-1}_5$ tells us that the indices in $\mathbf{a}_5$ of h4,8,9 are 4,0,6, we sort those indices, which gives 0,4,6. Then we order the haplotypes in $\mathbf{a}_{10}$ given these indices: 0 is h8 in $\mathbf{a}_5$, 4 is h4 in $\mathbf{a}_5$ and 6 is h9 in $\mathbf{a}_5$. Therefore the correct order should be h8,4,9 as generated by the sequential algorithm in Fig. S1. The haplotypes are now arranged correctly. The divergence values are fixed next.

The first divergence value is always correct (here 9), but the divergence values for h4 and h9 must be updated. For the second divergence (h4), $\mathbf{d_5}$ is scanned from scan_start = 1 to scan_stop = 4, the maximum value is 4 and is the correct divergence. For the third divergence (h9), d_5 is scanned from scan_start = 5 to scan_stop = 6, which is 0 (h4 and h9 are the same).

Now the group of three haplotypes is arranged correctly as h8,4,9 and the divergences values set to 9,4,0 have been corrected, and reflect the values shown in Fig. S1.

Algorithm 2 continues to go through $\mathbf{d_{10}}$ and encounters a divergence of 7 but the previous group is a single haplotype (h6), so we continue and encounter 8, 5, and finally 9. For the final divergence 9, the previous group is more than one haplotype, (h1, h2) so we have to correct their arrangement and divergence. Algorithm 1 is applied again and since h2 is before h1 in $\mathbf{a_5}$ they get rearranged as h2, h1. The first divergence of 8 is correct and the second one needs to be scanned in $\mathbf{d_5}$ and is found to be 1.

Finally, we check the last group. It is of size 1 (h5) so there is nothing to be done. Now the arrays $\mathbf{a_{10}}$ and $\mathbf{d_{10}}$ have been corrected using only the arrays $\mathbf{a_5}$ and $\mathbf{d_5}$ and reflect the expected values from the sequential version of Fig. S1.

This example shows how to generate the approximated **a** and **d** arrays in parallel, as well as the sequential correction steps to generate the same arrays as the sequential version but faster thanks to parallelism (the number of sequential correction steps is a constant).

Thanks to the two new algorithms it is possible to split a matrix of M haplotypes x N variant loci efficiently between T threads by first using T threads to compute approximated **a** and **d** arrays over regions of N/T=b variants and then sequentially fixing T-1 pairs of possibly erroneous arrays as shown in the example. After this step we have the required **a** and **d** arrays for the T threads to les each of them start processing one N_{th} of the whole matrix in parallel with the heavier algorithms (e.g., algorithm 3: Report Long Matches or algorithm 4: Report Set Maximal Matches (Durbin, 2014)).

Parallel execution of PBWT-based algorithms

A parallel execution of a PBWT-based algorithm is illustrated on Fig. SP1. The genomic region is split into chunks of size b (if the region is not a multiple of b, the last thread will simply cover a slightly smaller region). In order to launch the parallel processing each thread must already have the **a** and **d** arrays available for the start of their chunk. These arrays can be pre generated sequentially as shown in Fig. SP2.

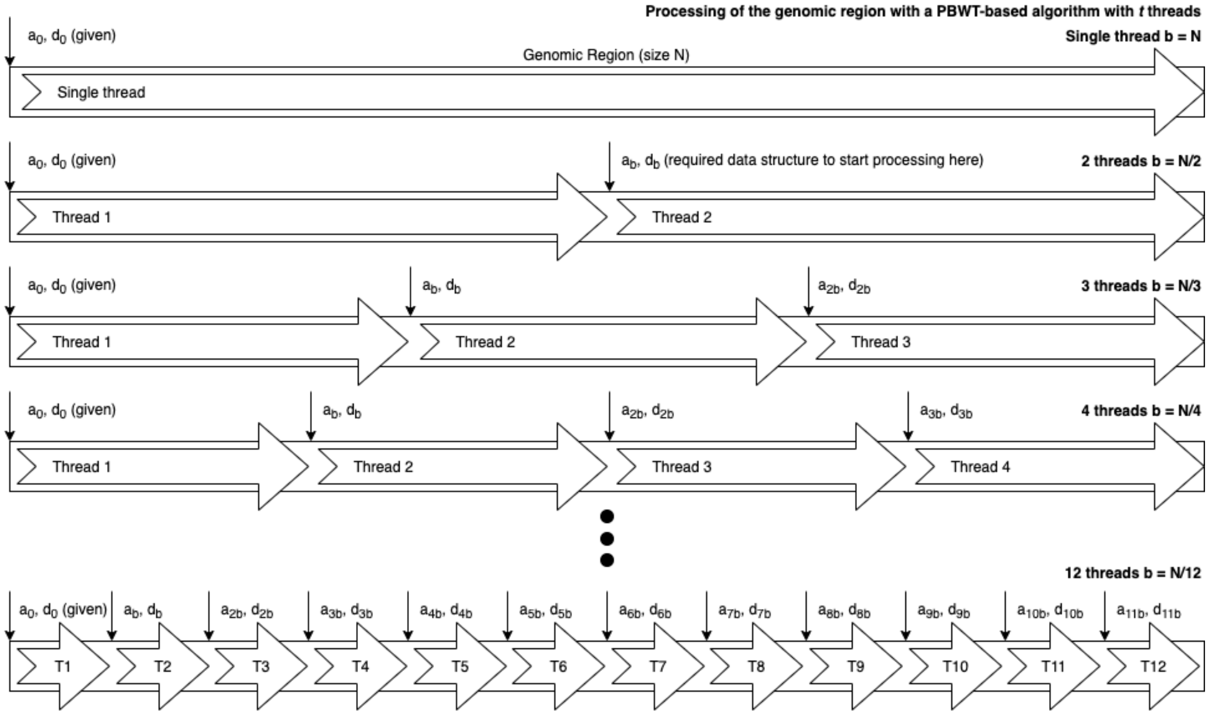

Fig SP1. Processing of a genomic region with a PBWT-based algorithm with different numbers of threads. All threads except the first one require generation of the **a** and **d** arrays for their starting position in order to start processing their chunk (of size b).

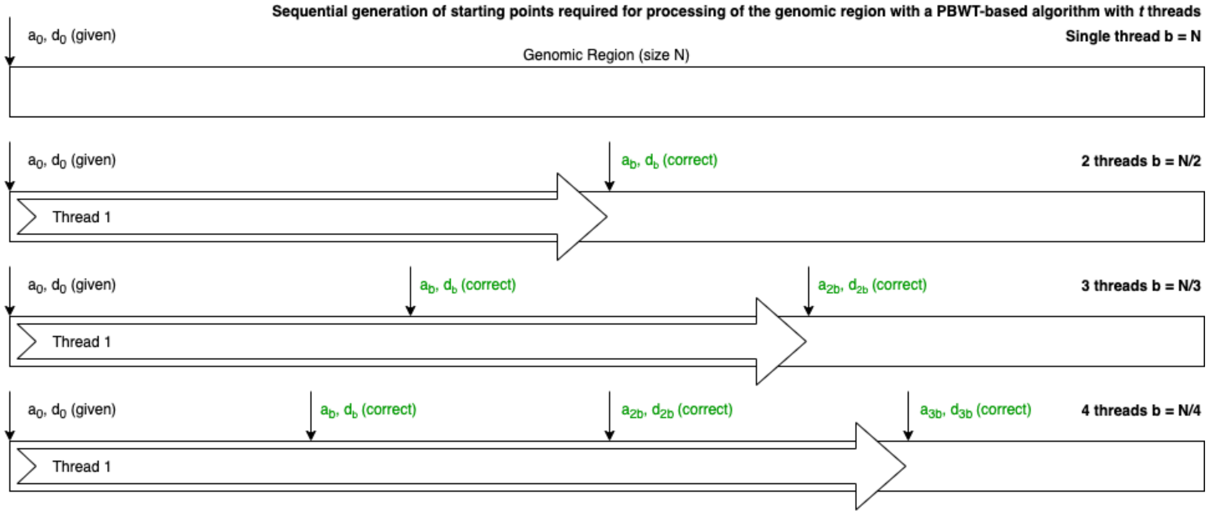

Fig SP2. The **a** and **d** arrays required can be generated sequentially by running algorithm 2 from (Durbin, 2014) and will therefore be correct.

However, approximated arrays can be generated in parallel with a number (t-1) threads starting with arbitrary **a** and **d** arrays as shown in Fig. SP3. Note that the first pair of arrays will always be correct because the initial **a** and **d** arrays are given.

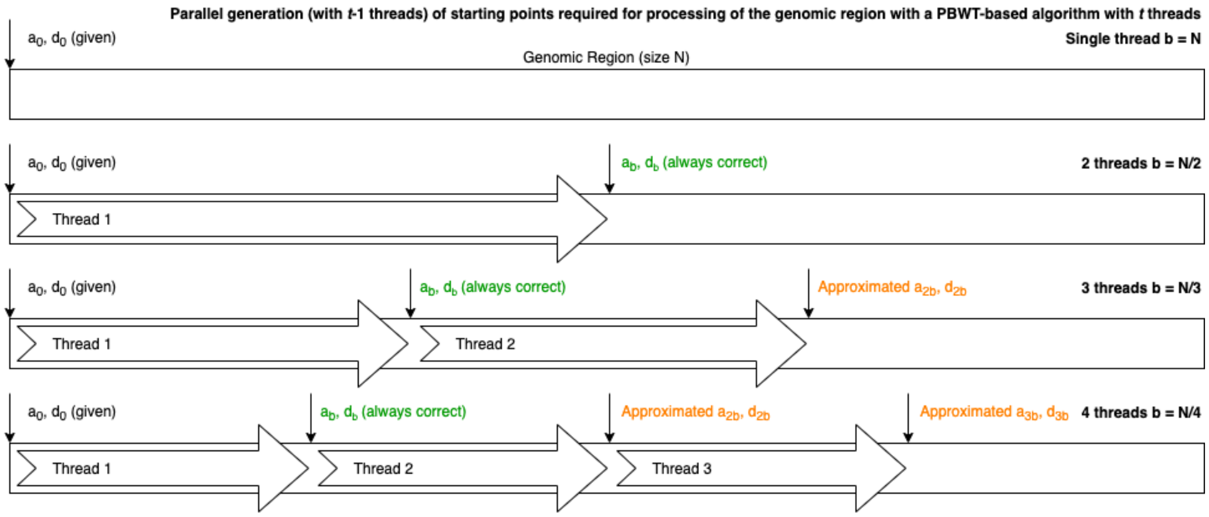

Fig SP3. Our approach allows us to generate approximated versions of the *a* and *d* arrays by running threads in parallel that will start with arbitrary arrays (see main text section 2.3).

The approximated arrays generated as shown in Fig. SP3 will need to be corrected in order to launch a parallel PBWT-based algorithm as shown in Fig. SP1. This can be done sequentially in a small number of steps as shown in Fig. SP4.

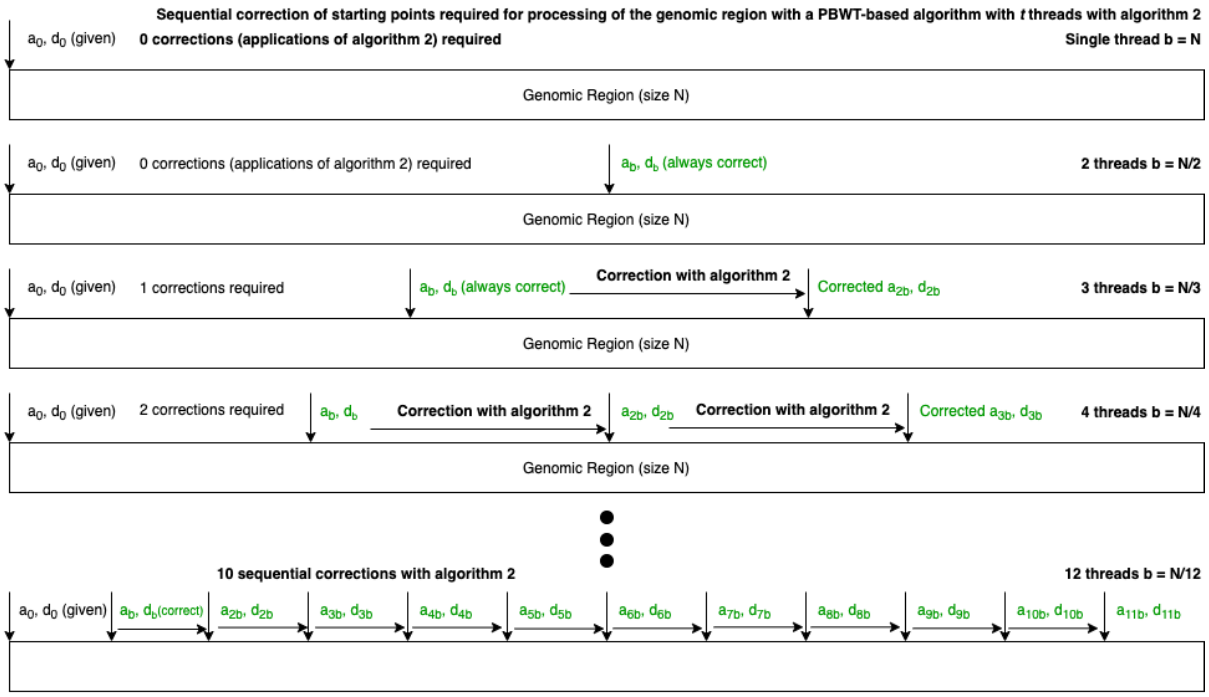

Fig SP4. The approximated arrays can be corrected effectively in a small number of sequential steps by applying algorithm 2 presented in the main text.

The correction of the approximated arrays is done by applying algorithm 2 presented in the main text. The number of steps required is small because the number of threads is small. The parallel generation followed by a sequential correction step is evaluated against the sequential approach (algorithm 2 of (Durbin, 2014)) below.

Comparison of sequential generation of arrays against parallel generation with sequential correction of arrays

The time required to generate the **a** and **d** arrays for parallel processing is shown in Fig. SP5. The parallel generation allows to reduce the processing time when more threads are used, even with the added computation cost of the sequential correction steps. The time required for the sequential generation of **a** and **d** arrays grows with the number of threads because it has to process a larger portion of the genomic region to generate the required *a* and *d* arrays as is illustrated in Fig. SP2.

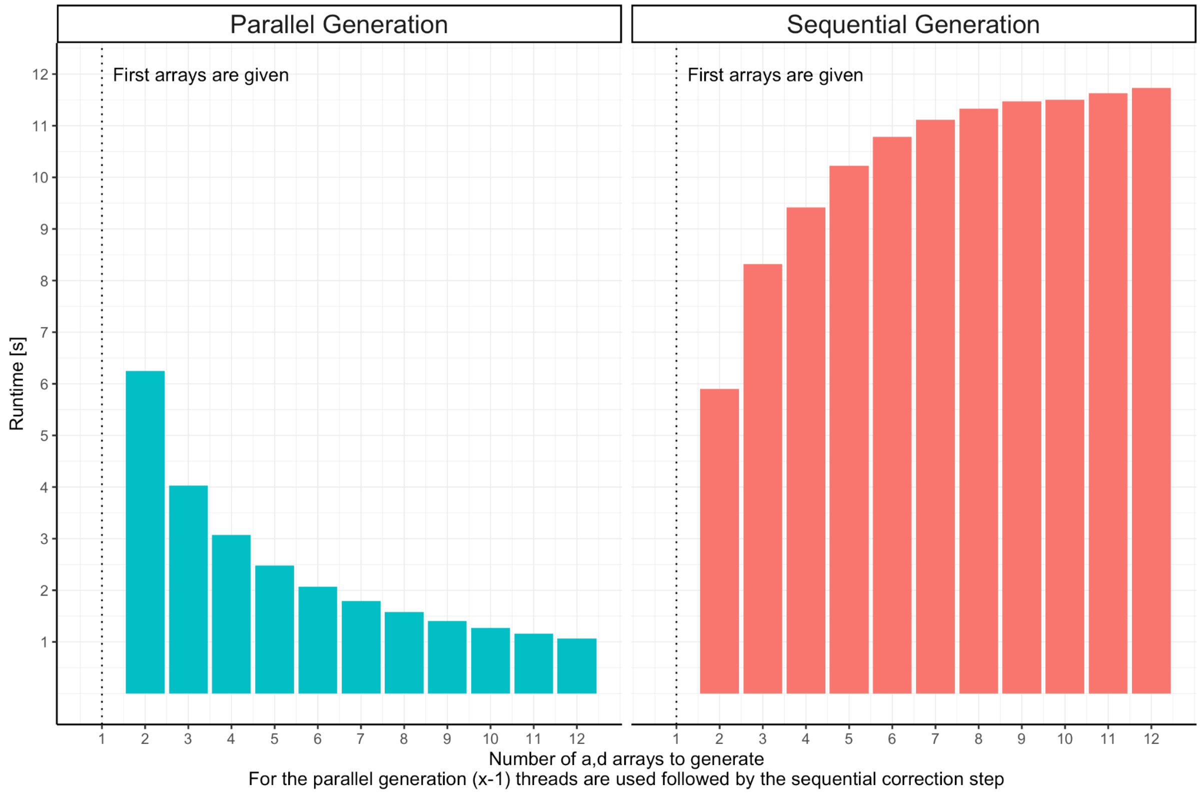

Fig SP5. The runtime of parallel generation of approximated **a** and **d** arrays with sequential correction step is shown on the left. The runtime of sequential generation of **a** and **d** arrays is shown on the right for different numbers of threads *t*. Input data is chromosome 20 from the One Thousand Genome Project Phase 3 (1KGP3) project (Consortium et al., 2015) (5,008 haplotypes, 1,822,268 genotypes).

The method proposed in the main text allows to generate the **a** and **d** arrays significantly faster than with the sequential approach (exhibiting a speed-up of **10.94x** when generating arrays for 12 threads). Note that once the **a** and **d** arrays are generated they could be saved to a file so that at a later time they can be used to apply PBWT-based algorithms in parallel directly without the need to recompute them. Also because the number of **a** and **d** arrays to store is small, they could be stored in a small index file that could be used to speed-up

subsequent analyses. For example, 64 arrays generated and saved to this file would allow to launch subsequent analysis with a number of threads up to 64 threads, but this would also allow to run algorithms with a lower number of threads e.g., 2, 4, or 12.

Extended Results

The original and multi-threaded versions of algorithm 3 and 4 (Durbin, 2014) were run on the Human Reference Consortium (HRC) (McCarthy et al., 2016) chromosome 20 (27,165 haplotypes, 884,697 genotypes). Results shown in Fig. S3 show a similar speed-up, up to 8 times as running the algorithms on the One Thousand Genome Project Phase 3 (1KGP3) (1000 Genomes Project Consortium and others, 2015) chromosome 20 (5,008 haplotypes, 1,822,268 genotypes). Algorithm 4 Report Set Maximal Matches shows some slow down with odd numbers of cores (3 and 11). This is suspected to be due to cache contention and uneven thread allocations on the 12-core CPU. Running with these odd amounts of threads is unrealistic, and even if there is a drop in speed compared to 2 and 10 threads, the speed-up compared to the base implementation. Algorithm 3 was run with a minimal matching length of 10,000 loci.

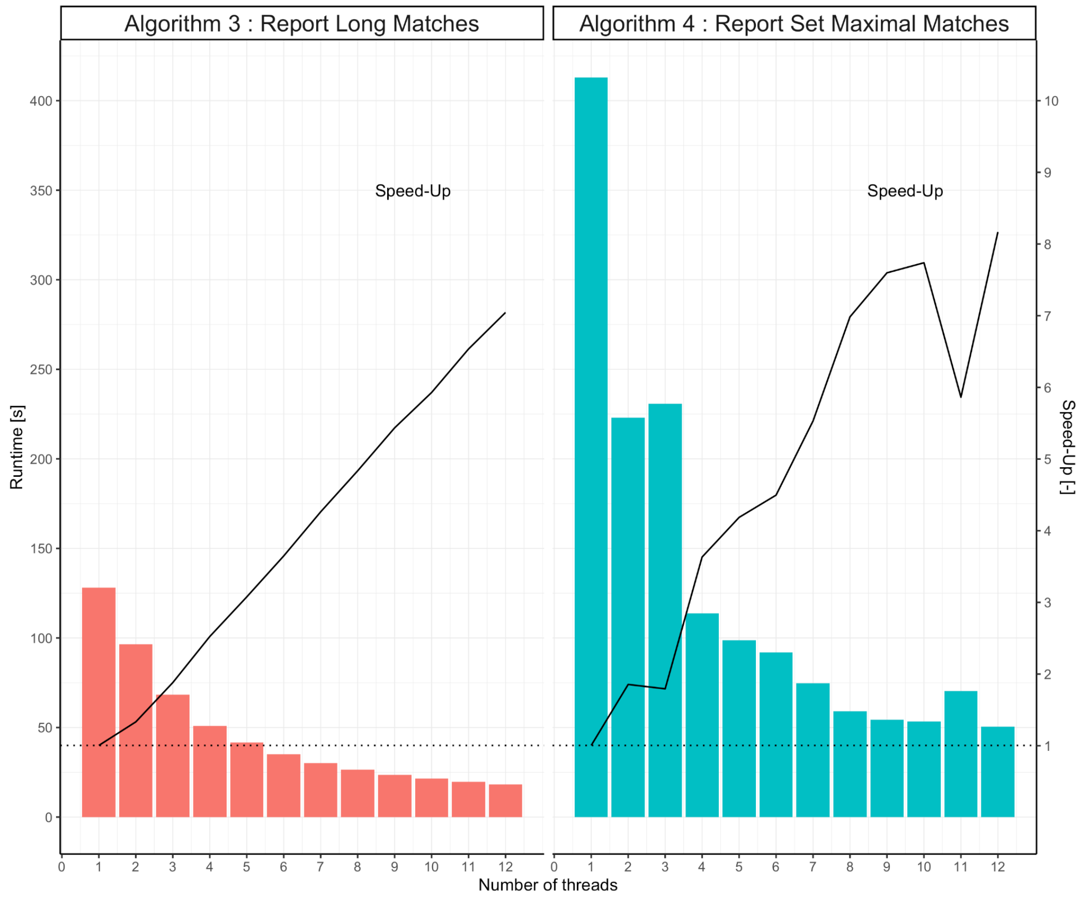

Fig. S3 Runtime and speed-up of algorithms 3 and 4 from (Durbin, 2014) and their parallel implementations running on a 12-core AMD 3900X processor. Algorithm 3 reports all matches longer than 10,000 genotypes between all haplotypes, and Algorithm 4 reports the set maximal matches between all haplotypes. Data is chromosome 20 from the Human Reference Consortium (McCarthy et al., 2016) (27,165 haplotypes, 884,697 genotypes).

In-application results

In order to quantify the speed-up in real case applications with file reading, processing, and writing output results to files, an application was written to implement the matching algorithms and is available on https://github.com/rwk-unil/parallel_pbwt. The application applies the parallel algorithms and provides the same results as the software from (Durbin, 2014) available from https://github.com/richarddurbin/pbwt (The results were compared empirically over the datasets). This allows us to compare the sequential and parallel versions in a more realistic setting. We ran algorithm 4 Report Set Maximal Matches on chromosome 20 from the 1000 Genome Project Phase 3 (5,008 haplotypes, 1,822,268 variant loci), both with the original PBWT software (Durbin, 2014) and our multi-threaded version. Both versions provide the same exact results.

The results are shown in Fig. S4. The total time running the original software is 102 [s], where 29 [s] are used to load the file and 73 [s] to run the algorithms and write the results to a file. With our multi-threaded implementation we can reduce the processing down to 38 [s] when running on 12 threads, resulting in a processing speed-up of almost **3x**. The reason why it only scales to 3x is because of the sequential input file reading and decompression (the input BCF file is gzip compressed). If we ignore the input file reading and decompression time (time which is given by both software) we achieve a speed-up of up to more than **7x** on the algorithm, including writing the results to files.

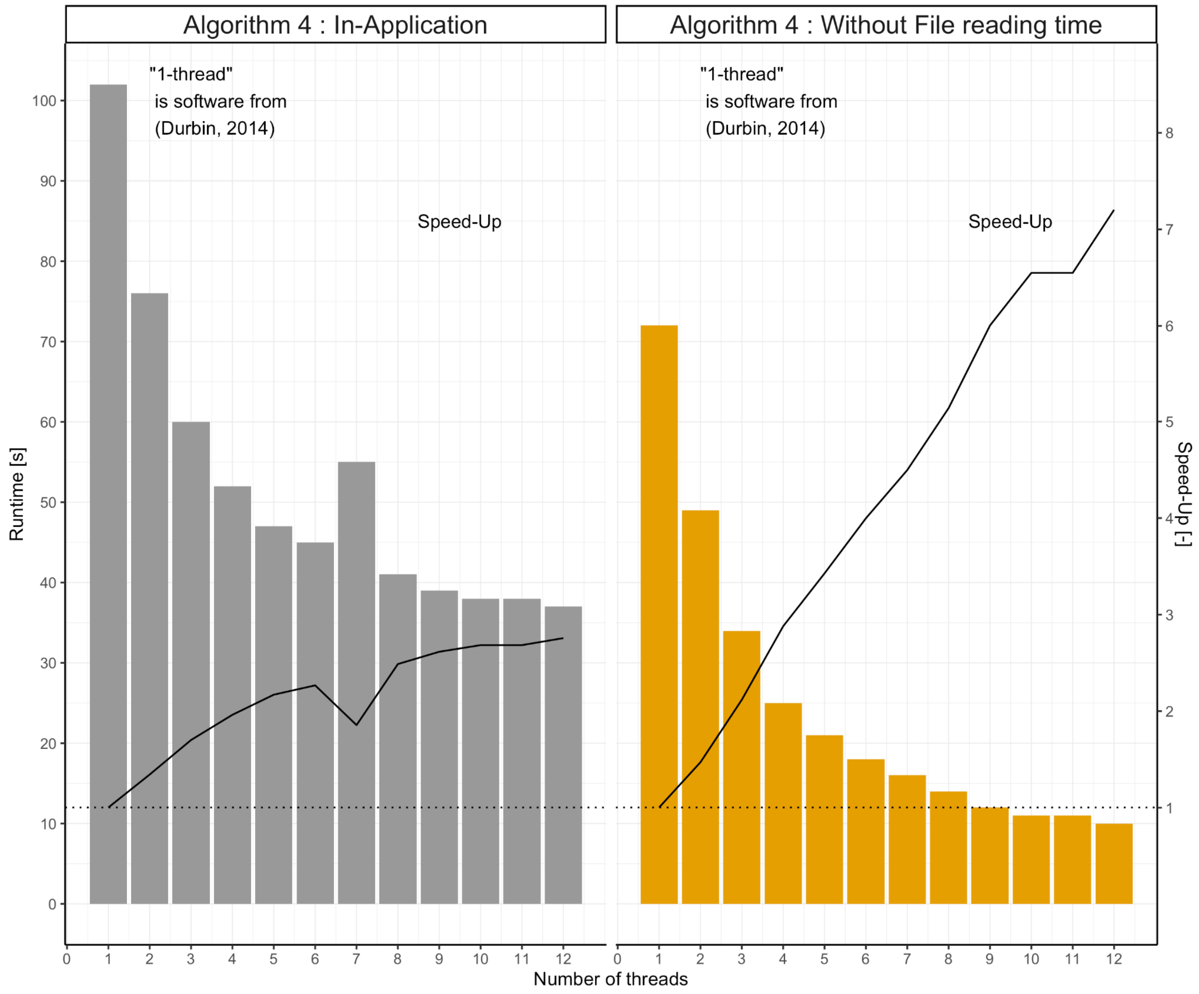

Fig. S4 In-application results of running Algorithm 4 Report Set Maximal Matches on chromosome 20 from the One Thousand Genome Project Phase 3 (1KGP3) project (Consortium et al., 2015) (5,008 haplotypes, 1,822,268 genotypes). Input file format is BCF. Left panel : Total runtime with input file reading, decompression, algorithm, writing results to output file. Right panel : Total runtime minus the input file reading and decompression. Input file reading is sequential and includes gzip decompression of the BCF file format.

The input file reading and decompression can be improved by switching to another file format such as XSI, which exhibits up to **8x** faster loading times as BCF for the same data (Wertenbroek et al., 2022).

References

- 1000 Genomes Project Consortium. (2015). A global reference for human genetic variation. *Nature*, *526*(7571), 68.
- Durbin, R. (2014). Efficient haplotype matching and storage using the positional Burrows–Wheeler transform (PBWT). *Bioinformatics*, *30*(9), 1266-1272.
- McCarthy, S., Das, S., Kretzschmar, W., Delaneau, O., Wood, A. R., Teumer, A., Kang, H. M., Fuchsberger, C., Danecek, P., Sharp, K., et al. (2016). A reference panel of 64,976 haplotypes for genotype imputation. Nature genetics, 48 (10), 1279.
- Wertenbroek, R., Rubinacci, S., Xenarios, I., Thoma, Y., & Delaneau, O. (2022). XSI—a genotype compression tool for compressive genomics in large biobanks. *Bioinformatics*, *38*(15), 3778-3784.
